# Impact of Medicaid Enrollment Timing on Tumor Stage at Diagnosis and Survival in Breast, Colorectal, and Lung Cancer

**DOI:** 10.3390/healthcare14060713

**Published:** 2026-03-11

**Authors:** Gabriel A. Benavidez, Stella Self, Anthony J. Alberg, Janice Probst, Jan M. Eberth

**Affiliations:** 1 Department of Public Health, Robbins College of Health and Human Sciences, Baylor University, Waco, TX 76798, USA; 2 Department of Epidemiology & Biostatistics, Arnold School of Public Health, University of South Carolina, Columbia, SC 29208, USA; scwatson@mailbox.sc.edu (S.S.); alberg@mailbox.sc.edu (A.J.A.); 3 Department of Health Services Policy & Management, Arnold School of Public Health, University of South Carolina, Columbia, SC 29208, USA; probst.rural@gmail.com; 4 Department of Population Health Sciences, Virginia-Maryland College of Veterinary Medicine, Virginia Tech, Blacksburg, VA 24061, USA; jmeberth@vt.edu

**Keywords:** Medicaid enrollment, breast cancer, colorectal cancer, lung cancer, survival, tumor stage, policy

## Abstract

**Background:** Medicaid-insured patients experience higher rates of late-stage cancer diagnosis and worse survival than non-Medicaid patients. The impact of Medicaid enrollment timing on cancer outcomes is less clear. This study examines the association between Medicaid enrollment and timing with tumor stage and cancer-specific survival for breast, colorectal, and lung cancers. **Methods:** We analyzed SEER-Medicaid linked data for 276,755 breast, 104,784 colorectal, and 101,058 lung cancer patients < 65 years of age. Patients were categorized as non-Medicaid enrollees, pre-diagnosis enrollees (≥12 months before), or post-diagnosis enrollees (≤12 months after). Multivariable logistic regression estimated odds ratios of late-stage diagnosis, and cause-specific Cox proportional hazards models were used to assess cancer-specific survival, adjusting for demographic and socioeconomic factors. **Results:** Compared to non-Medicaid enrollees, post-diagnosis enrollees had the highest odds of late-stage diagnosis (breast cancer: OR: 3.41; colorectal cancer: OR: 3.78; lung cancer: OR: 1.87). Pre-diagnosis enrollees also had increased odds, but the association was weaker than post-diagnosis enrollees. Cancer-specific mortality was higher for both pre- and post-diagnosis enrollees compared to non-Medicaid enrollees for each cancer examined across tumor stage at diagnosis. Among Medicaid enrollees, those enrolled post-diagnosis had higher cancer-specific mortality than those enrolled pre-diagnosis for localized-stage colorectal (HR: 1.82) and lung cancer (HR: 1.30). In contrast, those enrolled post-diagnosis had lower mortality than those enrolled pre diagnosis for distant-stage breast cancer (HR: 0.91). **Conclusions:** Compared with cancer patients not insured by Medicaid, post-diagnosis Medicaid enrollment was associated with a greater likelihood of late-stage cancer and worse cancer-specific survival across each cancer type examined. Future research is warranted to examine the role of Medicaid enrollment timing in cancer care to better understand its impact on cancer outcomes.

## 1. Introduction

In the United States, a lack of or inadequate health insurance is one of the most consistent barriers to receiving timely healthcare services [1]. In 2023, approximately 25 million Americans (9.5% of the population) had no form of health insurance [2]. This is especially troublesome for cancer prevention and control efforts, as early detection and treatment depends on timely access to healthcare services. The early detection and timely treatment of cancers is one of the most effective mechanisms for decreasing the burden of cancer morbidity and mortality as cancers detected and treated in earlier stages are more responsive to treatments and typically have a favorable prognosis compared with malignancies diagnosed in later stages [3]. This is particularly critical for lung, breast, and colorectal cancers, which together account for nearly 40% of new cancer cases and cancer-related deaths each year in the U.S. [4]. Given their high morbidity and mortality burden, identifying modifiable policy-level factors that influence tumor stage at diagnosis and cancer-specific survival may help identify policy strategies to increase access to healthcare and improve outcomes for patients with lung, breast, and colorectal cancers.

The Medicaid program provides free or low-cost health insurance coverage to an estimated 83 million eligible low-income Americans, with eligibility determined by federal minimum standards and additional criteria that vary by state [5,6]. Previous work has shown that in states that expanded their Medicaid eligibility criteria, providing coverage to individuals who would have previously been uninsured, states saw increased detection of earlier stage cancers and improved cancer survival [7,8]. However, when comparing cancer outcomes such as tumor stage at diagnosis and cancer survival across insurance coverage types, those with Medicaid coverage remain more likely to have tumors diagnosed at later stages and experience worse survival compared to those with private insurance [9,10,11]. This suggests that access to and quality of healthcare services for Medicaid beneficiaries, compared to those with private insurance, remains lacking and may contribute to worse cancer outcomes. Notably, due to many states’ varying eligibility requirements, many patients may only be eligible to enroll in Medicaid after a cancer diagnosis, which could result in missed opportunities for earlier cancer detection and thus improved survival [12]. While past research has consistently linked Medicaid insurance to a higher likelihood of advanced-stage cancer and lower survival compared to non-Medicaid insurance, the role of Medicaid enrollment timing on these outcomes remains less clear.

The timing of Medicaid enrollment likely influences cancer outcomes through access pathways: specifically, continuous pre-diagnosis coverage supports the uptake of routine screening and primary care, reduces diagnostic delays, and facilitates timely treatment, leading to earlier stage at diagnosis and better survival, whereas enrollment only at or after diagnosis or with coverage disruptions increases the risk of late presentation and worse outcomes. Evidence already exits to suggest that delayed Medicaid enrollment may be particularly detrimental. Bradley et al. (2005) [13] found that individuals who enrolled in Medicaid only after a cancer diagnosis had significantly worse survival than those enrolled pre diagnosis, likely due to delayed access to screening and early treatment. More recently, Bradley et al. (2021) [14] reinforced this finding, showing that compared to pre-diagnosis Medicaid enrollees, post-diagnosis Medicaid enrollees were more likely to present with late-stage disease and had higher cancer-specific mortality risks across multiple cancers. Similarly, Xie et al. (2022) [15] found that compared to post-diagnosis Medicaid enrollment, earlier Medicaid enrollment was associated with a lower likelihood of late-stage breast cancer diagnosis, with survival benefits primarily observed among those with sustained Medicaid coverage. Despite recent insights, research on Medicaid enrollment timing remains limited, often constrained to single-state studies or individual cancer types, leaving gaps in understanding how delays in coverage affect cancer outcomes across diverse populations. To address this gap, this study utilizes a nationally representative Surveillance Epidemiology and End Results (SEER)-Medicaid dataset to examine the association between Medicaid enrollment timing with tumor stage at diagnosis and mortality for breast, colorectal, and lung cancers.

## 2. Methods

### 2.1. Data Source and Study Design

This study used a SEER-Medicaid linked dataset, which combines data from 14 SEER cancer registries (2006–2013) with Medicaid Analytic Extract (MAX) files for the same period [16,17]. SEER provides comprehensive cancer incidence data, while Medicaid MAX includes enrollment records from all 50 states and Washington, D.C. A deterministic matching process linked individuals across datasets, identifying 559,484 Medicaid enrollees from 3,493,820 total SEER cancer cases.

The SEER-Medicaid files for breast, colorectal, and lung cancers included 632,309, 347,049, and 450,486 mutually exclusive patients in each dataset, respectively. A priority for this analysis was to examine cancer outcomes among non-elderly patients; therefore, persons ≥ 65 years were excluded as were non-primary cancer cases and those cancer cases found at autopsy. By limiting our study population to those who were <65 years, we greatly reduce the proportion of people who are also insured through Medicare, a distinct federal program from Medicaid that provides health insurance for those ≥ 65 years and younger persons with certain disabilities.

A cross-sectional study design was used to examine tumor stage at diagnosis, assessing Medicaid enrollment status relative to cancer detection. For survival outcomes, a retrospective cohort study design with prospective follow-up was applied, following patients diagnosed from 2006 to 2013 through 2018 to evaluate cancer-specific survival. The maximum follow-up period was 12–13 years, depending on diagnosis year. Given that this study used deidentified secondary data from the SEER-Medicaid database and did not involve human subjects, it is considered exempt from IRB review.

### 2.2. Classification of Medicaid Enrollment Status

Our primary independent variable was Medicaid status classified according to being enrolled or not and, if enrolled, enrollment timing (pre- or post-cancer diagnosis). Using prior research on this topic as a guide [13], data from SEER and Medicaid enrollment files were used to classify patients into three groups: not enrolled in Medicaid, enrolled within 12 months pre-diagnosis, or enrolled the month of or within 12 months post-diagnosis.

### 2.3. Study Outcomes

The two dependent variables were tumor stage at diagnosis and survival. Tumor stage at diagnosis was determined using SEER summary stages (in situ, localized, regional, and distant). Consistent with prior studies [14,15,18], we dichotomized tumor stage into early-stage diagnosis (in-situ and localized) and late-stage diagnosis (regional and distant). Overall survival and cancer-specific survival was assessed using the prespecified variables in the SEER-Medicaid dataset, which provides a vital status variable based on International Classification of Disease (ICD) codes. Survival was measured in months from cancer diagnosis to the last follow-up or recorded death, whichever occurred first. Follow-up for vital status extended through 2018.

### 2.4. Covariates

We also examined common demographic variables, such as age, sex, census tract level income, race/ethnicity, and rural–urban residency. Age (in years) was a continuous measure provided by SEER. For confidentiality purposes, some variables are only provided at the census tract level. Median income was a variable restricted to only the census tract level. Race and ethnicity were determined from patient SEER data derived from hospital records and were categorized as Hispanic, Non-Hispanic Black, Non-Hispanic White, and Non-Hispanic Other. Rurality was determined based on the county of residence for the patient at their time of diagnosis and defined using 2013 rural–urban continuum codes [19]. Rural–urban continuum codes (RUCC) range from 1 to 9 with 1 being the least rural and 9 being the most rural. In line with previous work, we categorized RUCCs 1–3 as urban and 4–9 as rural [20].

### 2.5. Statistical Analysis

We examined differences in demographic and socioeconomic variables across Medicaid status and enrollment timing (not enrolled in Medicaid, enrolled pre-diagnosis, and enrolled after diagnosis) using medians for continuous variables (age and census tract median income) and proportions for categorical variables (rurality and race/ethnicity). Statistical significance among covariates across insurance status groups was assessed using the Kruskal–Wallis test for continuous variables and chi-square tests for categorical variables.

To assess the association between insurance status/timing and tumor stage at diagnosis, we employed multivariable mixed effects regression models. Adjusted models incorporated age, sex (for colorectal and lung cancers), rural–urban residency, and census-tract-level median income. We included a random intercept for state to account for unobserved state-level variation and patient clustering, and a likelihood ratio test supported the inclusion of this random effect. Because the dataset includes cases from 14 SEER population-based cancer registries, which are not nested within states (some registries are statewide while others represent single metropolitan areas), we adjusted for registry differences by including SEER registry fixed effects, implemented as indicator variables for each registry. This approach permits control for registry-specific case mix and reporting differences without imposing an incorrect hierarchical data structure. Analyses were conducted separately for breast, colorectal, and lung cancer patients.

We compared cumulative incidence of cancer-specific death across insurance status and timing using Gray’s test to account for competing risks. We then fit a cause-specific Cox proportional hazards model to assess the association between insurance status and timing and cancer-specific mortality, interpreting effects on the cause-specific hazard among individuals at risk. A state-level frailty term was incorporated to account for unobserved heterogeneity across states. SEER registry fixed effects were also included to adjust for registry-level differences. Model fit was evaluated using the Akaike information criterion (AIC), which supported this specification for each cancer type. The proportional hazards assumption was examined and was violated for tumor stage at diagnosis; therefore, survival analyses were stratified by tumor stage (localized, regional, distant). All analyses were performed using SAS version 9.4.

## 3. Results

### 3.1. Sample Demographic Characteristics and Group Differences

The analytic cohort included 276,755 breast cancer patients, 104,784 colorectal cancer patients, and 101,058 lung cancer patients. The median age in years was 51, 53, and 56 for breast, colorectal, and lung cancer patients, respectively. Across all cancer types, the majority of patients were non-Hispanic White, resided in urban counties, and were non-Medicaid enrollees during the year before their cancer diagnosis. Most breast (95.6%) and colorectal (76.4%) cancer patients were diagnosed with early-stage disease, whereas the reverse was true for lung cancer patients, with 65.2% diagnosed with late-stage disease (Table 1).

Significant differences in demographic characteristics were observed between non-Medicaid enrollees, pre-diagnosis enrollees, and post-diagnosis enrollees for each cancer type (Table 2). Compared with non-Medicaid enrollees, pre-diagnosis Medicaid enrollees had median census tract incomes that were 18.9% lower for breast cancer ($49,956 vs. $59,388), 15.1% lower for colorectal cancer ($47,941 vs. $55,194), and 14.4% lower for lung cancer ($46,553 vs. $53,257). Across all cancer types, a higher proportion of racial and ethnic minority patients were observed in the pre-diagnosis and post-diagnosis Medicaid enrollee groups compared to non-Medicaid enrollees. For example, among breast cancer patients, 10.9% of non-Medicaid enrollees identified as Hispanic, whereas the proportion was 20.1% among pre-diagnosis enrollees and 21.8% among post-diagnosis enrollees.

### 3.2. Tumor Stage at Diagnosis

#### 3.2.1. Breast Cancer

After adjusting for demographic and socioeconomic factors, compared to non-Medicaid enrollees, the odds of being diagnosed with late-stage breast cancer were significantly higher among pre-diagnosis enrollees (OR: 2.05, 95% CI: 1.93–2.16) and even higher among post-diagnosis enrollees (OR: 3.41, 95% CI: 3.23–3.58). Furthermore, post-diagnosis enrollees were significantly more likely to be diagnosed with late-stage disease compared to pre-diagnosis enrollees (OR: 1.62, 95% CI: 1.51–1.72) (Table 3).

#### 3.2.2. Colorectal Cancer

Among colorectal cancer patients, compared to non-Medicaid enrollees, the odds of being diagnosed with late-stage colorectal cancer were significantly higher among pre-diagnosis enrollees (OR: 1.43, 95% CI: 1.39–1.52) and even higher among post-diagnosis enrollees (OR: 3.78, 95% CI: 3.59–3.93). Furthermore, post-diagnosis enrollees were significantly more likely to be diagnosed with late-stage disease compared to pre-diagnosis enrollees (OR: 2.59, 95% CI: 2.44–2.76) (Table 3).

#### 3.2.3. Lung Cancer

In contrast to breast and colorectal cancer patients, pre-diagnosis enrollees with lung cancer did not have significantly higher odds of being diagnosed with late-stage lung cancer compared to non-Medicaid enrollees (OR: 0.99, 95% CI: 0.96–1.02). However, the odds of a late-stage diagnosis were significantly higher among post-diagnosis enrollees compared to non-Medicaid enrollees (OR: 1.87, 95% CI: 1.80–1.96). Additionally, post-diagnosis enrollees had significantly higher odds of late-stage disease compared to pre-diagnosis enrollees (OR: 1.85, 95% CI: 1.77–1.93) (Table 3).

### 3.3. Cancer Specific Survival Outcomes

#### 3.3.1. Breast Cancer

For localized breast cancer cases, compared to non-Medicaid enrollees, mortality risk was significantly higher among pre-diagnosis enrollees (HR: 2.11, 95% CI: 1.96–2.26) and post-diagnosis enrollees (HR: 2.09, 95% CI: 1.93–2.26), with a nearly identical increase in risk (Table 4). There was no difference in mortality risk between pre-diagnosis and post-diagnosis enrollees for localized disease (HR: 1.00, 95% CI: 0.91–1.10) (Table 4).

For regional-stage breast cancer, a similar pattern was observed, with pre-diagnosis enrollees (HR: 1.76, 95% CI: 1.67–1.84) and post-diagnosis enrollees (HR: 1.68, 95% CI: 1.60–1.76) experiencing a higher mortality risk compared to non-Medicaid enrollees. Again, there was no significant difference in mortality risk between pre-diagnosis enrollees and post-diagnosis enrollees (HR: 0.96, 95% CI: 0.91–1.02).

For distant-stage breast cancer cases, compared to non-Medicaid enrollees, mortality risk was significantly higher among pre-diagnosis enrollees (HR: 1.42, 95% CI: 1.34–1.51) and post-diagnosis enrollees (HR: 1.29, 95% CI: 1.22–1.36). However, in contrast to localized and regional disease, post-diagnosis enrollees had a significantly lower mortality risk compared to pre-diagnosis enrollees (HR: 0.91, 95% CI: 0.85–0.98).

#### 3.3.2. Colorectal Cancer

Among localized colorectal cancer cases, compared to non-Medicaid enrollees, mortality risk was significantly higher among pre-diagnosis enrollees (HR: 1.92, 95% CI: 1.74–2.13) and post-diagnosis enrollees (HR: 3.50, 95% CI: 3.09–3.97), with post-diagnosis enrollees experiencing the greatest risk (Table 4). Mortality risk was also significantly higher for post-diagnosis enrollees compared to pre-diagnosis enrollees (HR: 1.82, 95% CI: 1.59–2.13) (Table 4).

For regional-stage colorectal cancer, a similar pattern was observed, with pre-diagnosis enrollees (HR: 1.61, 95% CI: 1.52–1.71) and post-diagnosis enrollees (HR: 2.00, 95% CI: 1.89–2.12) experiencing a higher mortality risk compared to non-Medicaid enrollees. Mortality risk was also significantly higher among post-diagnosis enrollees compared to pre-diagnosis enrollees (HR: 1.25, 95% CI: 1.16–1.35).

Among distant-stage colorectal cancer cases, compared to non-Medicaid enrollees, mortality risk was significantly higher among pre-diagnosis enrollees (HR: 1.36, 95% CI: 1.30–1.42) and post-diagnosis enrollees (HR: 1.25, 95% CI: 1.20–1.29). However, there was no significant difference in mortality risk between pre-diagnosis and post-diagnosis enrollees (HR: 0.93, 95% CI: 0.90–1.04).

#### 3.3.3. Lung Cancer

Among localized lung cancer cases, compared to non-Medicaid enrollees, mortality risk was significantly higher among pre-diagnosis enrollees (HR: 1.76, 95% CI: 1.63–1.89) and post-diagnosis enrollees (HR: 2.27, 95% CI: 2.03–2.53), with post-diagnosis enrollees experiencing the highest risk (Table 4). Mortality risk was also significantly higher for post-diagnosis enrollees compared to pre-diagnosis enrollees (HR: 1.30, 95% CI: 1.15–1.45). (Table 4)

For regional-stage lung cancer, a similar trend was observed, with pre-diagnosis enrollees (HR: 1.32, 95% CI: 1.27–1.38) and post-diagnosis enrollees (HR: 1.50, 95% CI: 1.43–1.57) experiencing a higher mortality risk compared to non-Medicaid enrollees. Mortality risk was also significantly higher among post-diagnosis enrollees compared to pre-diagnosis enrollees (HR: 1.14, 95% CI: 1.08–1.20).

Among distant-stage lung cancer cases, compared to non-Medicaid enrollees, mortality risk was significantly higher among pre-diagnosis enrollees (HR: 1.16, 95% CI: 1.14–1.19) and post-diagnosis enrollees (HR: 1.11, 95% CI: 1.08–1.13). However, there was no significant difference in mortality risk between pre-diagnosis and post-diagnosis enrollees (HR: 0.97, 95% CI: 0.93–1.03).

## 4. Discussion

This study examined the association between Medicaid status and enrollment timing with tumor stage at diagnosis and cancer-specific survival in breast, colorectal, and lung cancers. Across all three cancers, Medicaid enrollment was associated with a higher prevalence of late-stage diagnosis and worse survival outcomes, with post-diagnosis Medicaid enrollees experiencing the poorest outcomes, particularly for localized and regional-stage colorectal and lung cancers.

For breast and colorectal cancers, both pre- and post-diagnosis enrollees had higher odds of late-stage diagnosis than non-Medicaid patients. However, for lung cancer, only post-diagnosis enrollees had increased odds, potentially due to limited screening recommendations during the study period.

In cancer-specific survival, Medicaid enrollees had worse outcomes than non-enrollees, with post-diagnosis enrollees at higher risk than pre-diagnosis enrollees in localized and regional colorectal and lung cancers, suggesting factors beyond late-stage detection contribute to survival differences. Interestingly, we observed that breast cancer patients who enrolled in Medicaid after their cancer diagnosis had better survival outcomes than those enrolled prior to diagnosis. One possible explanation for this counterintuitive finding is the role of the National Breast and Cervical Cancer Early Detection Program (NBCCEDP) [21], which facilitates diagnostic services and care coordination for low-income women. Through this program, women diagnosed with breast cancer may become eligible for Medicaid post-diagnosis via the Breast and Cervical Cancer Treatment Program. As such, these post-diagnosis enrollees may receive more timely and structured care than typically observed in other cancers, potentially mitigating the negative effects often associated with post-diagnosis Medicaid enrollment.

The notable increase in mortality risk among post-diagnosis Medicaid enrollees highlights the importance of early Medicaid enrollment in potentially reducing late-stage diagnoses and improving survival outcomes. Previous studies have shown that Medicaid-insured patients consistently experience worse cancer outcomes than privately insured patients, potentially due to differences in early detection [22,23] and treatment access [24]. Given that many uninsured individuals qualify for Medicaid but remain unenrolled, policy efforts that prioritize early outreach, automatic enrollment mechanisms, and streamlining administrative procedures to facilitate access to coverage before a cancer diagnosis hold promise for improving cancer outcomes.

Our results, in combination with previous research [9,25,26,27], reinforce the disparities in both tumor stage at diagnosis and cancer-specific survival experienced by Medicaid-insured patients. One major contributing factor is differences in preventive screening uptake. A national study [28] demonstrated that screening rates for cervical, breast, and colorectal cancers were significantly lower among Medicaid beneficiaries compared to individuals with commercial insurance. While our dataset lacked screening information, previous studies have established that better screening adherence is associated with earlier-stage diagnosis and improved survival outcomes.

The unique findings regarding lung cancer tumor stage at diagnosis highlight the potential role of screening policies. Unlike breast and colorectal cancer, national lung cancer screening guidelines by the United States Preventive Service Task Force were not widely implemented during our study period (2006–2013) [29], which may explain why prior Medicaid enrollment did not reduce late-stage diagnosis compared to non-enrollees. More recent evidence suggests that since the introduction of national lung cancer screening guidelines in 2013 [29], the proportion of early-stage diagnoses has increased across all insurance types, albeit less for Medicaid enrollees compared to private insurance [30]. These findings underscore the need for future research to determine whether Medicaid enrollees are fully benefiting from lung cancer screening initiatives or if barriers to early detection persist in this population.

The association between post-diagnosis Medicaid enrollment and higher mortality risk suggests that expanding Medicaid eligibility alone may not be sufficient to reduce cancer disparities if enrollment delays persist. Prior research by Bradley et al. (2005) [13] found similar patterns in breast, colorectal, and lung cancers, emphasizing that Medicaid patients—regardless of enrollment timing—experience inferior survival. Our study expands on this work by also directly comparing prior and post-diagnosis Medicaid enrollment and demonstrating that, while earlier Medicaid enrollment is beneficial, Medicaid patients overall still face survival disadvantages compared to non-Medicaid populations.

Our findings are relevant to Medicaid policy discussions. While expanding Medicaid eligibility increases healthcare coverage, a significant number of eligible individuals remain uninsured due to administrative and procedural barriers. In 2022, an estimated 6.4 million (<65 years old) nonelderly individuals—25% of the uninsured population—were eligible for Medicaid or the Children’s Health Insurance Program but not enrolled, often due to difficulties navigating the application process or procedural disenrollments [31]. To strengthen cancer control efforts, policies that prioritize outreach and enrollment initiatives to address these barriers are needed to help ensure Medicaid-eligible individuals receive timely coverage and preventive services before a cancer diagnosis occurs. Furthermore, existing low-cost screening programs, such as the National Breast and Cervical Cancer Early Detection Program (NBCCEDP), remain underutilized [21]. Reports indicate that approximately 60% of eligible women do not receive regular screenings through this program, representing missed opportunities to detect cancers earlier and improve survival rates [21]. Expanding the reach and accessibility of such programs, including expanding the program to include other types of cancer screening (i.e., colorectal and lung cancer screening), could significantly reduce late-stage diagnoses and improve outcomes for low-income populations.

### Strengths and Limitations

This study is among only a few that have used the SEER-Medicaid dataset to examine the impact of Medicaid enrollment timing on tumor stage at diagnosis and survival across breast, colorectal, and lung cancers in a large, national sample. Prior research has been limited to single states, healthcare centers, or single cancers, making this analysis a significant contribution to understanding insurance-related disparities across multiple cancer types.

However, multiple limitations must be acknowledged. First, we lack data on screening practices, which likely mediate the relationship between Medicaid enrollment timing and tumor stage. Thus, our results capture the total effect rather than the direct effect of Medicaid status on tumor stage at diagnosis, which remains an important indicator. Second, the SEER-Medicaid dataset lacks individual-level socioeconomic data (e.g., income, education), requiring reliance on area-level measures, which are useful but less precise. Third, because the dataset spans 2006–2013, it predates Medicaid expansion, meaning findings may differ with more recent data that account for state-level expansion status.

A key limitation of this study relates to state-level variation in Medicaid eligibility pathways, which complicates the inference related to the effect of enrollment timing. In several states, a cancer diagnosis itself can trigger new eligibility. For example, programs such as the National Breast and Cervical Cancer Early Detection Program (NBCCEDP) and medically needy/spend-down provisions allow uninsured individuals to qualify for Medicaid only after receiving a diagnosis. As a result, some people we classify as post-diagnosis enrollees may not have been eligible for Medicaid prior to their cancer diagnosis, and therefore their late enrollment reflects policy structure rather than delayed engagement with the healthcare system. At the same time, a distinct subgroup of individuals may have been fully eligible for Medicaid before diagnosis but remained unenrolled due to administrative barriers, limited awareness, stigma, or difficulty navigating the enrollment process. These individuals also enter our dataset as post-diagnosis enrollees, even though their delayed coverage represents unmet need rather than policy-driven ineligibility. Because our data cannot distinguish between those newly eligible due to a cancer diagnosis and those eligible but unenrolled prior to diagnosis, our post-diagnosis category combines conceptually different populations. This misclassification may attenuate or bias associations between enrollment timing and cancer outcomes, since the mechanisms producing late enrollment differ and may carry different risks for late-stage presentation and mortality.

Finally, our non-Medicaid enrollee group most likely includes both privately insured and uninsured individuals, which may bias associations toward the null. Because uninsured patients typically present with more advanced disease and have worse survival than privately insured patients, grouping them together may compress outcome differences between Medicaid and the comparison group and bias our effect estimates toward the null. Specifically, if the uninsured subgroup is at substantially higher risk than the privately insured, the average of the two obscures meaningful contrasts, and the true magnitude of Medicaid’s association with earlier diagnosis and improved survival may be larger than we report. Future work in this area may benefit from further stratification by insurance type to better isolate Medicaid’s relative effects. Additionally, results of this study are limited to the U.S. context and may not be transportable to other countries with different healthcare systems.

## 5. Conclusions

Our findings underscore the persistent disparities in tumor stage at diagnosis and survival for Medicaid beneficiaries compared to non-Medicaid populations. Importantly, these disparities are driven in part by late or unstable coverage, as many individuals gain Medicaid coverage only at or after diagnosis or experience coverage disruptions, patterns linked to advanced stage and worse outcomes. Expanding Medicaid eligibility and increasing enrollment uptake among eligible but uninsured individuals may help reduce these disparities. Policies that not only expand eligibility but also focus on timely uptake among eligible but uninsured individuals, while reducing administrative barriers, minimizing coverage turnover, and ensuring access to high-quality cancer care, are critical to narrowing disparities. Future research should evaluate how more recent Medicaid expansion policies and evolving screening guidelines have changed the role of Medicaid enrollment timing on cancer outcomes among the Medicaid insured population.

## Figures and Tables

**Table 1:** SEER-Medicaid dataset (2006–2013) patient characteristics by cancer type.

Variables	Breast Cancer (n = 276,555)	Colorectal Cancer (n = 104,784)	Lung Cancer (n = 101,058)
**Median Age, Years**	51.0	53.0	56.0
**Census Tract Median Income**	$57,500	$54,100	$52,100
**Sex**			
Male	-	55.9	55.14
Female	100	44.1	44.9
**Race/Ethnicity**			
Hispanic	12.5	12.8	6.2
Non-Hispanic White	64.7	61.7	71.5
Non-Hispanic Black	12.0	14.6	15.4
Non-Hispanic Other	10.8	10.9	6.8
**County Rurality**			
Urban	91.18	87.8	83.9
Rural	8.8	12.2	16.1
**Medicaid Enrollment Status**			
Not Enrolled	83.8	79.8	66.2
Enrolled Prior	8.6	11.4	19.8
Enrolled Post	7.6	8.6	14.0
**Tumor Stage at Diagnosis**			
Early Stage	95.6	76.4	34.8
Late Stage	4.4	23.6	65.2

**Table 2:** SEER-Medicaid dataset (2006–2013) patient characteristics across Medicaid enrollment status and timing categories for breast, colorectal, and lung cancers.

	Breast Cancer				Colorectal Cancer				Lung Cancer			
	Not Enrolled (n = 231,915)	Prior (n = 23,742)	Post (n = 20,898)	*p*-Value	Not Enrolled (n = 83,833)	Prior (n = 11,966)	Post (n = 8985)	*p*-Value	Not Enrolled (n = 66,893)	Prior (n = 19,985)	Post (n = 14,180)	*p*-Value
**Median Age, Years**	52.0	50.0	52.0	<0.001	53.0	52.0	54.0	<0.001	56.0	55.0	54.0	<0.001
**Census Tract Median Income**	$59,400	$49,900	$51,700	<0.001	$55,200	$47,900	$49,300	<0.001	$53,300	$46,600	48,700	<0.001
**Sex**												
Male	-	-	-		56.4	49.21	60.06	<0.001	54.66	52.89	60.59	<0.001
Female	100	100	100		43.6	50.79	39.94		45.34	47.11	39.41	
**Race/Ethnicity**												
Hispanic	10.9	20.1	21.8	<0.001	11.6	18.0	17.4	<0.001	5.84	6.79	7.35	<0.001
Non-Hispanic White	68.5	45.0	44.2		65.1	47.6	47.9		75.71	63.27	63.17	
Non-Hispanic Black	9.7	25.6	22.3		12.2	24.7	24.9		11.1	25.17	22.22	
Non-Hispanic Other	10.9	9.3	11.7		11.1	9.7	9.8		7.35	4.77	7.26	
**County Rurality**												
Urban	91.96	87.07	87.22	<0.001	88.7	83.51	85.02	<0.001	86.14	78.61	81.19	<0.001
Rural	8.04	12.93	12.78		11.3	16.49	14.98		13.86	21.39	18.81	
**Tumor Stage at Diagnosis ^a^**												
Early Stage	96.57	92.66	88.68	<0.001	79.68	72.83	50.62	<0.001	36.62	36.68	23.66	<0.001
Late Stage	3.43	7.34	11.32		20.32	27.17	49.38		63.38	63.32	76.34	
**Median Survival, Months**	141.0	120.0	117.0	<0.001	113.0	83.0	64.0	<0.001	40.0	26.0	22.0	<0.001

^a^ Early-stage includes in situ and localized tumors, while late-stage includes regional and distant SEER-staged tumors.

**Table 3:** Adjusted * odds ratios for the association between Medicaid enrollment status and timing with late-stage cancer diagnosis in SEER-Medicaid patients (2006–2013).

Medicaid Status & Enrollment Timing	Adjusted OR (95% CI)
**Breast Cancer**	
Not Enrolled (Reference)	1.00
Enrolled Prior to Diagnosis vs. Not Enrolled	2.05 (1.93–2.16) **
Enrolled Post-Diagnosis vs. Not Enrolled	3.41 (3.23–3.58) **
Enrolled Prior to Diagnosis (Reference)	1.00
Enrolled Post-Diagnosis vs. Enrolled Prior to Diagnosis	1.62 (1.51–1.72) **
**Colorectal Cancer**	
Not Enrolled (Reference)	1.00
Enrolled Prior to Diagnosis vs. Not Enrolled	1.43 (1.39–1.52) **
Enrolled Post-Diagnosis vs. Not Enrolled	3.78 (3.59–3.93) **
Enrolled Prior to Diagnosis (Reference)	1.00
Enrolled Post-Diagnosis vs. Enrolled Prior to Diagnosis	2.59 (2.44–2.76) **
**Lung Cancer**	
Not Enrolled (Reference)	1.00
Enrolled Prior to Diagnosis vs. Not Enrolled	0.99 (0.96–1.02)
Enrolled Post-Diagnosis vs. Not Enrolled	1.87 (1.80–1.96) **
Enrolled Prior to Diagnosis (Reference)	1.00
Enrolled Post-Diagnosis vs. Enrolled Prior to Diagnosis	1.85 (1.77–1.93) **

* Adjusted models include the following variables: Age, sex (colorectal/lung only), rurality, census tract median income. ** Indicates statistical significance at α = 0.05.

**Table 4:** Adjusted * hazard ratios for Medicaid enrollment status and timing with cancer-specific mortality in SEER-Medicaid patients (2006–2013), stratified by tumor stage at diagnosis, with follow-up through 2018.

	Tumor Stage at Diagnosis		
	Localized HR (95% CI)	Regional HR (95% CI)	Distant HR (95% CI)
**Breast Cancer**			
Not Enrolled (Reference)	1.00	1.00	1.00
Enrolled Prior vs. Not Enrolled	2.11 (1.96, 2.26)	1.76 (1.67, 1.84)	1.42 (1.34, 1.51)
Enrolled Post vs. Not Enrolled	2.09 (1.93, 2.26)	1.68 (1.60, 1.76)	1.29 (1.22, 1.36)
Enrolled Prior (Reference)	1.00	1.00	1.00
Enrolled Post vs. Enrolled Prior	1.00 (0.91, 1.10)	0.96 (0.91, 1.02)	0.91 (0.85, 0.98)
	**Tumor Stage at Diagnosis**		
**Colorectal Cancer**	**Localized** **HR (95% CI)**	**Regional** **HR (95% CI)**	**Distant** **HR (95% CI)**
Not Enrolled (Reference)	1.00	1.00	1.00
Enrolled Prior vs. Not Enrolled	1.92 (1.74, 2.13)	1.61 (1.52, 1.71)	1.36 (1.30, 1.42)
Enrolled Post vs. Not Enrolled	3.50 (3.09, 3.97)	2.00 (1.89, 2.12)	1.25 (1.20, 1.29)
Enrolled Prior (Reference)	1.00	1.00	1.00
Enrolled Post vs. Enrolled Prior	1.82 (1.59, 2.13)	1.25 (1.16, 1.35)	0.93 (0.90, 1.04)
	**Tumor Stage at Diagnosis**		
**Lung Cancer**	**Localized** **HR (95% CI)**	**Regional** **HR (95% CI)**	**Distant** **HR (95% CI)**
Not Enrolled (Reference)	1.00	1.00	1.00
Enrolled Prior vs. Not Enrolled	1.76 (1.63, 1.89)	1.32 (1.27, 1.38)	1.16 (1.14, 1.19)
Enrolled Post vs. Not Enrolled	2.27 (2.03, 2.53)	1.50 (1.43, 1.57)	1.11 (1.08, 1.13)
Enrolled Prior (Reference)	1.00	1.00	1.00
Enrolled Post vs. Enrolled Prior	1.30 (1.15, 1.45)	1.14 (1.08, 1.20)	0.97 (0.93, 1.03)

* Adjusted models include the following variables: age, sex (colorectal/lung only), rurality, census tract median income.

## Data Availability

The authors cannot make the data publicly available because of the restrictions of the SEER-Medicaid Data Use Agreement. Researchers can visit https://healthcaredelivery.cancer.gov/seermedicaid/ (1 May 2022) for information about requesting and accessing the SEER-Medicaid data.
